# Screening for abdominal aortic aneurysm

**DOI:** 10.1590/S1516-31802004000400001

**Published:** 2004-07-01

The incidence of abdominal aortic aneurysm (AAA) has been increasing over the last fifty years, probably as a consequence of the smoking epidemics that developed in the years that followed the Second World War. As stated by Puech-Leão et al., abdominal aortic aneurysm is an asymptomatic but potentially fatal condition, for which the proper treatment is elective surgery before rupturing can occur.^1^

The Multicentre Aneurysm Screening Study (MASS) was designed to assess whether or not such screening is beneficial. A population-based sample of men (n = 67,800) aged 65-74 years was enrolled, and each individual was randomly allocated to either receive an invitation for an abdominal ultrasound scan (invited group; n = 33,389) or not (control group; n = 33,961). The primary outcome measurement was mortality related to abdominal aortic aneurysm. Of the 33,389 men invited to participate in screening, 27,147 accepted the invitation and 1,333 aneurysms were detected. Using mortality data from the Office of National Statistics, an intention-to-treat analysis was performed, based on cause of death. There were 65 aneurysm-related deaths (absolute risk of 0.0019) in the screened group, compared with 113 (absolute risk of 0.0033) in the control group (absolute risk reduction of 0.014), with a number needed to screen of 714. What does this mean? It means that we have to screen 714 people to prevent one death related to an AAA.^2^

Screening people has positive and negative consequences. In the case of screening for an aortic aneurysm, the positive result is the prevention of a possible rupture of the aneurysm. The negative result is to label an asymptomatic individual as a sick man. In the MASS Study, four scales were used for assessing quality of life. Six weeks after screening, there were no differences in anxiety or depression between those who screened negative and those who screened positive. There were just small differences in health status measurements: those who screened positive had slightly lower scores on the physical and mental subscales of SF-36, and lower self-assessments of their health, as measured by EuroQol.^2^ Results from other study have shown that screening for AAA results in impairment of quality of life among those who have the disease and were suffering from poor quality of life prior to screening. Among those who had an age-adjusted good quality of life prior to screening and were found to have the disease, and among those who were found to have normal aortas, no negative effect on quality of life was observed. The study concluded that low quality of life before screening is a possible risk factor for negative mental effects in diagnosing an AAA by screening.^3^

## What can we offer to an individual with an AAA detected by screening?

We now have a large body of evidence recommending surgery for aneurysms of diameter 5.5 cm. In the UK Small Aneurysm Trial, 1,090 participants aged 60-76 years with a symptomless abdominal aortic aneurysm of 4.0-5.5 cm in diameter were randomly assigned to undergo early elective open surgery (n = 563) or ultrasonographic surveillance (n = 527). Patients were followed up for an average of 4.6 years. Surgical repair was recommended if the diameter of the aneurysms in the surveillance group exceeded 5.5 cm. The hazard ratio for all-cause mortality in the surveillance group was 0.94 (95% confidence interval [CI]: 0.75-1.17). It was concluded that ultrasonographic surveillance for small abdominal aortic aneurysm is safe, but early surgery does not provide a long-term survival advantage.^4^

## Who needs to be screened?

Lederle et al. studied the prevalence of independent risk factors for abdominal aortic aneurysm in a cross-sectional study on 75,451 war veterans who were aged 50-79 years and had no previous history of AAA. Smoking was the risk factor most strongly associated with AAA: the association increased significantly with the number of years of smoking and decreased significantly with the number of years after quitting smoking. The excess prevalence associated with smoking accounted for 78% of all AAA that were 4.0 cm or larger in this study. Other independently associated risk factors included age, height, presence of coronary heart disease, any atherosclerosis, high cholesterol levels and hypertension.^5^

## What is the cost-effectiveness of screening for AAA?

Wilmink et al. studied the cost-effectiveness of screening for AAA. Screening was estimated to have prevented 10.8 ruptured AAA and 8 deaths per year, thereby gaining 51 life-years per year for the study population, and to have reduced the incidence of ruptured AAA by 64% (95% CI: 42%-77%). Each life-year gained during the first screening round cost US$ 1107. To save one life, 1,000 men needed to be screened and 5 elective operations performed. The cost-effectiveness of screening for AAA depends on the number of elective surgeries in the population screened. Most aneurysms detected by screening are smaller and should be kept under surveillance with periodic ultrasonographic measurement.^6^ The widespread elective repair of small AAA could reduce the benefits and increase the costs of screening.^7^

## What screening method can we use for detecting an AAA?

The gold-standard method for AAA diagnosis is the ultrasonographic measurement. However, several studies have evaluated the sensitivity and specificity of abdominal palpation for detecting AAA. Pooled data from 15 studies have shown that the sensitivity of abdominal palpation increases significantly with AAA diameter (p < 0.01), ranging from 29% for an AAA of 3.0 to 3.9 cm to 76% for an AAA of 5.0 cm or greater. The positive and negative likelihood ratios with 95% confidence intervals that were found when using a cutoff point for AAA size of 3.0 cm or greater were 12.0 (95% CI: 7.4-19.5) and 0.72 (95% CI: 0.65-0.81), respectively.^8^ Fink et al. studied 99 subjects with an AAA diagnosed by previous ultrasonography and 101 without any AAA diagnosis. All the patients were submitted to abdominal palpation by two internists who were blind to the results of the ultrasound. The sensitivity of the abdominal palpation was 68% (95% CI: 6-%-76%), with specificity of 75% (95% CI: 68%-82%), positive likelihood ratio of 2.7 (95% CI: 2.0-3.6) and negative likelihood ratio of 0.43 (95% CI: 0.33-0.56). The inter-observer pair agreement between the first and second observers was 77% (kappa value of 0.53). The sensitivity of the palpation increased with AAA diameter, and reached 82% for AAA of 5.0 cm or larger. The sensitivity was also greater among individuals with an abdominal girth of less than 100 cm.^9^

In the paper by Puech-Leão et al.,^1^ the prevalence of AAA among the population of São Paulo aged over 50 years is between 1.80 and 2.96%. However, among men who are older than 60 years, it is estimated to be 4.34 to 7.95% and will probably be even higher among currently smoking men over the age of 60. Data from this study show that there are a lot of discrepancies between the results from abdominal palpation and ultrasound. However, these discrepancies probably decreased at the same type as the AAA diameter increased.

It is very important to teach the advantages and disadvantages of various maneuvers that can be utilized in physical examinations. Abdominal palpation is not a perfect tool for diagnosing an AAA. However, epidemiological reasoning, in association with technical training in abdominal palpation, could enhance our chances of detecting an AAA. Thus, for men aged 60 years or more who have at some time in their lives smoked, abdominal palpation for AAA detection and maybe an ultrasound ought to be incorporated into routine clinical examinations.
